# Outbreak of HIV Infection Linked to Nosocomial Transmission, China, 2016–2017

**DOI:** 10.3201/eid2412.180117

**Published:** 2018-12

**Authors:** Xiaohong Pan, Jianmin Jiang, Qiaoqin Ma, Jiafeng Zhang, Jiezhe Yang, Wanjun Chen, Xiaobei Ding, Qin Fan, Zhihong Guo, Yan Xia, Shichang Xia, Zunyou Wu

**Affiliations:** These first authors contributed equally to this articleZhejiang Provincial Center for Disease Control and Prevention, Hangzhou, China (X. Pan, J. Jiang, Q. Ma, J. Zhang, J. Yang, W. Chen, X. Ding, Q. Fan, Z. Guo, Y. Xia, S. Xia);; National Center for AIDS/STD Control and Prevention, Beijing, China (Z. Wu);; University of California, Los Angeles, California, USA (Z. Wu)

**Keywords:** Human immunodeficiency virus, HIV, viruses, nosocomial transmission, lymphocyte immunotherapy, China

## Abstract

On January 25, 2017, a physician from ZC Hospital in Hangzhou, China, reported to the Zhejiang Provincial Center for Disease Control and Prevention that a potential HIV outbreak might have occurred during lymphocyte immunotherapy (LIT) performed at the hospital on December 30, 2016. We immediately began investigating and identified the index case-patient as an LIT patient’s husband who donated lymphocytes for his wife’s LIT and later screened HIV-reactive. Subsequent contamination by a technician resulted in the potential exposure of 34 LIT patients. Acute HIV infection was diagnosed in 5 persons. Phylogenetic analysis confirmed that the HIV-1 *gag*, *pol*, and *env* gene sequences from the index and outbreak-related cases had >99.5% similarity. Rapid investigation and implementation of effective control measures successfully controlled the outbreak. This incident provides evidence of a lapse in infection control causing HIV transmission, highlighting the need for stronger measures to protect patients from infectious disease exposure.

Lymphocyte immunotherapy (LIT) to treat recurrent miscarriage involves receipt of lymphocytes to a patient from a donor, usually the patient’s male partner. Although the European Society of Human Reproduction and Embryology (*1*), the Royal College of Obstetricians (*2*), and the American College of Obstetricians and Gynecologists (*3*) have issued clear guidance against LIT, supported by a 2014 Cochrane review (*4*), more recent meta-analyses support its use (*5**,**6*), as do 4 newer intervention control studies conducted in China (*7**–**10*). Although the number of LIT recipients in China is estimated to be large, no statistics are available. Within China’s healthcare system, LIT is a category III medical service, meaning that each hospital regulates itself (*11*).

On January 24, 2017, a woman receiving LIT at ZC Hospital in Hangzhou, China, called a hospital staff member, Dr. X, asking if she had risk for HIV infection. She explained that her husband had just received a confirmed diagnosis of HIV infection and that on December 30, 2016, she had received LIT using lymphocytes her husband donated. Dr. X immediately reported this information to the hospital’s deputy director, who informed the clinical medical laboratory director, Dr. Y. At ≈4:00 pm the same day, Dr. Y informed the responsible laboratory technician, Dr. Z, and requested that she stop LIT. One hour later, Dr. Z voluntarily reported to Dr. Y that she had deviated from protocol on December 30 and that other patients who received LIT on the same day might have been exposed. At 5:30 pm, the director of ZC Hospital called an emergency meeting with department directors, who decided to request help from the Zhejiang Provincial Center for Disease Control and Prevention (Zhejiang CDC). On January 25, 2017, Zhejiang CDC epidemiologists began investigating a possible HIV outbreak among LIT recipients at ZC Hospital. We report on the investigation conducted, control measures implemented, and outcomes observed.

## Methods

The potential HIV outbreak at ZC Hospital was declared a public health emergency, and a formal investigation began on January 25, 2017, supported by provincial (Zhejiang Health Commission and Zhejiang CDC) and national (National Health Commission and National Center for AIDS/STD Control and Prevention, Chinese Center for Disease Control and Prevention [China CDC]) authorities and resources. Neither institutional review board approval nor individual informed consent was required for the investigation. Routine informed consent for HIV, hepatitis B virus (HBV), hepatitis C virus (HCV), and syphilis testing (oral or written) and for contact tracing (oral) was obtained.

### Case Definition, Case Finding, and Contact Tracing

We defined an outbreak-related case as a newly diagnosed laboratory-confirmed HIV infection, with evidence of acute infection suggesting occurrence of transmission on December 30, 2016, among women who had received LIT at ZC Hospital that day or their secondary contacts, with HIV gene sequence highly related to that of the index case-patient. Initial case finding began among all women who received LIT at ZC Hospital on December 30. A trained public health specialist conducted interviews on HIV risk behavior during December 30, 2016–January 25, 2017, to assess the possibility that HIV infection had been acquired by means other than LIT and that HIV already had been transmitted to others.

### HIV, HBV, HCV, and Syphilis Testing

All potential outbreak-related case-patients and their contacts were provided free testing and counseling at ZC Hospital. Persons in whom HIV infection was diagnosed were referred to treatment. For HIV, serologic screening was conducted at ZC Hospital’s laboratory using the Anti-HIV (1+2) 4th-generation antigen/antibody enzyme immunoassay (EIA) kit (Shanghai Kehua Bio-Engineering, Shanghai, China) and the HIV 1/2/O Tri-Line HIV Rapid Test Device (ABON Biopharm, Hangzhou). If reactive, new venous blood specimens were collected and sent to the Hangzhou Center for Disease Control and Prevention laboratory for confirmatory serologic testing by Western blot (WB; MP Biomedicals, Singapore). In parallel, plasma specimens were sent to the Zhejiang CDC, where HIV nucleic acid testing was conducted using COBAS AmpliPrep/COBAS TaqMan HIV-1 Test v2.0 kits (Roche, Branchburg, NJ, USA).

HBV, HCV, and syphilis testing were performed at ZC Hospital’s laboratory. For HBV, samples were screened for 5 indicators (i.e., hepatitis B surface antigen, hepatitis B surface antibody, hepatitis B e-antigen, hepatitis B e-antibody, hepatitis B c-antibody) using EIA kits (InTec Products, Xiamen, China). For HCV, samples were screened for antibodies using an EIA kit (Zhuhai Livzon Diagnostics, Zhuhai, China). For syphilis, samples were initially screened by Toluidine Red Untreated Serum Test (TRUST, Shanghai Rongsheng Biotech, Shanghai, China). Reactive samples were confirmed by *Treponema pallidum* particle agglutination assay (Fujirebio Inc., Nagasaki, Japan).

### Laboratory Audit

An audit of the hospital laboratory began immediately on January 25 and lasted 6 days. It was conducted by 3 trained compliance specialists and 3 public health officials from independent institutions. They thoroughly reviewed all relevant records: staffing, training, qualification, certification, security, inventory, equipment, LIT protocol, compliance, supervision, and infection control procedures, as well as records generated during the execution of LIT-related procedures. The audit also included private interviews with all laboratory staff and direct observation of staff rehearsing LIT procedures; investigation of other potential violations of protocol that might have caused nosocomial transmission; and a check of baseline laboratory tests for HIV, HBV, HCV, and syphilis for all 34 women and their husbands.

### Molecular Phylogeny Analysis

All HIV sequencing was performed at the Zhejiang CDC laboratory using plasma specimens. Two technicians in separate laboratory areas extracted HIV RNA from specimens, each using a different method: one used the QIAamp Viral RNA Mini Kit (QIAGEN, Hilden, Germany), the other a viral RNA/DNA extraction kit on an automatic extraction platform (Suzhou Tianlong, Suzhou, China). Partial sequences for the HIV genes *gag*, *pol*, and *env* were amplified by reverse transcription PCR and nested PCR using GUX/GDX primers for *gag*, 5 different pairs of primers for *pol*, and M13F/M13R primers for *env*. PCR products were confirmed by 1% agarose gel electrophoresis and then purified and sequenced.

We analyzed sequences with Sequencher v5.0 (Gene Codes Corporation, Ann Arbor, MI, USA), examined them for similarity, and aligned them to reference sequences using BioEdit v7.2.0 (Ibis Therapeutics, Carlsbad, CA, USA). Two sets of reference sequences for each gene were selected for comparison to outbreak-related consensus sequences. The first set was international reference sequences obtained from the Los Alamos National Laboratory (https://www.hiv.lanl.gov). The second was representative of strains circulating in the area at the time of the outbreak. We used the neighbor-joining tree method (Kimura 2-parameter model) to determine HIV subtype and phylogenetic relationships and genetic distance between sequences. Two technicians blindly and independently analyzed 2 specimens from each patient. Neighbor-joining phylogenetic trees were constructed using MEGA 6.0 (https://www.megasoftware.net) with 1,000 replicate bootstrap alignments. We defined a transmission cluster as having a bootstrap value >90% and a mean genetic distance of <0.015.

## Results

### Epidemiologic Investigation

Mrs. P0, age 36, is the wife of the index-case patient, P0, and had been enrolled in LIT starting June 21, 2016. At enrollment, P0 and Mrs. P0 both underwent behavioral health screening, physical examination, and HIV testing (both tested HIV negative). Mrs. P0 received LIT on July 19, August 16, September 13, October 14, November 11, December 2, and December 30, 2016, at ZC Hospital, each time with lymphocytes donated by her husband 3 days before her LIT dates. Later in the day after her December 30 treatment, Mrs. P0 learned that her husband had screened HIV reactive. On December 31, Mrs. P0 went to XX Hospital in Hangzhou, which treats persons living with HIV, where she informed the physician she had just discovered she was pregnant and had great concern about possible exposure to HIV by LIT because her husband had just screened HIV-reactive, which she worried she might transmit to her unborn baby. She was strongly encouraged to immediately begin postexposure prophylaxis (PEP), using a regimen of 3 antiretroviral medications (tenofovir, lopinavir/ritonavir, and lamivudine) for 4 weeks. She started PEP the same day.

Upon attending antenatal care shortly thereafter, Mrs. P0 tested negative for HIV, HBV, HCV, and syphilis. On January 24, 2017, after her husband received a confirmed diagnosis of HIV infection, Mrs. P0 alerted staff at ZC Hospital that she was concerned she was exposed to HIV via LIT on December 30, 2016; this report was the initiating event that sparked the outbreak investigation. As part of the investigation, Mrs. P0 was followed and regularly tested for HIV, HBV, HCV, and syphilis. All results were repeatedly negative. Her PEP regimen was converted to a prevention of mother-to-child transmission (PMTCT) program (same medicines extended to 8 weeks after delivery).

Mrs. P0’s baby was born on July 9, 2017, and Mrs. P0 stopped all use of antiretroviral therapy (ART) on the same day. Her most recent HIV screening test was performed on March 9, 2018, at 9 months since she stopped ART and >14 months since her exposure. Her results remained negative.

### Index Case-Patient

**Figure 1:**
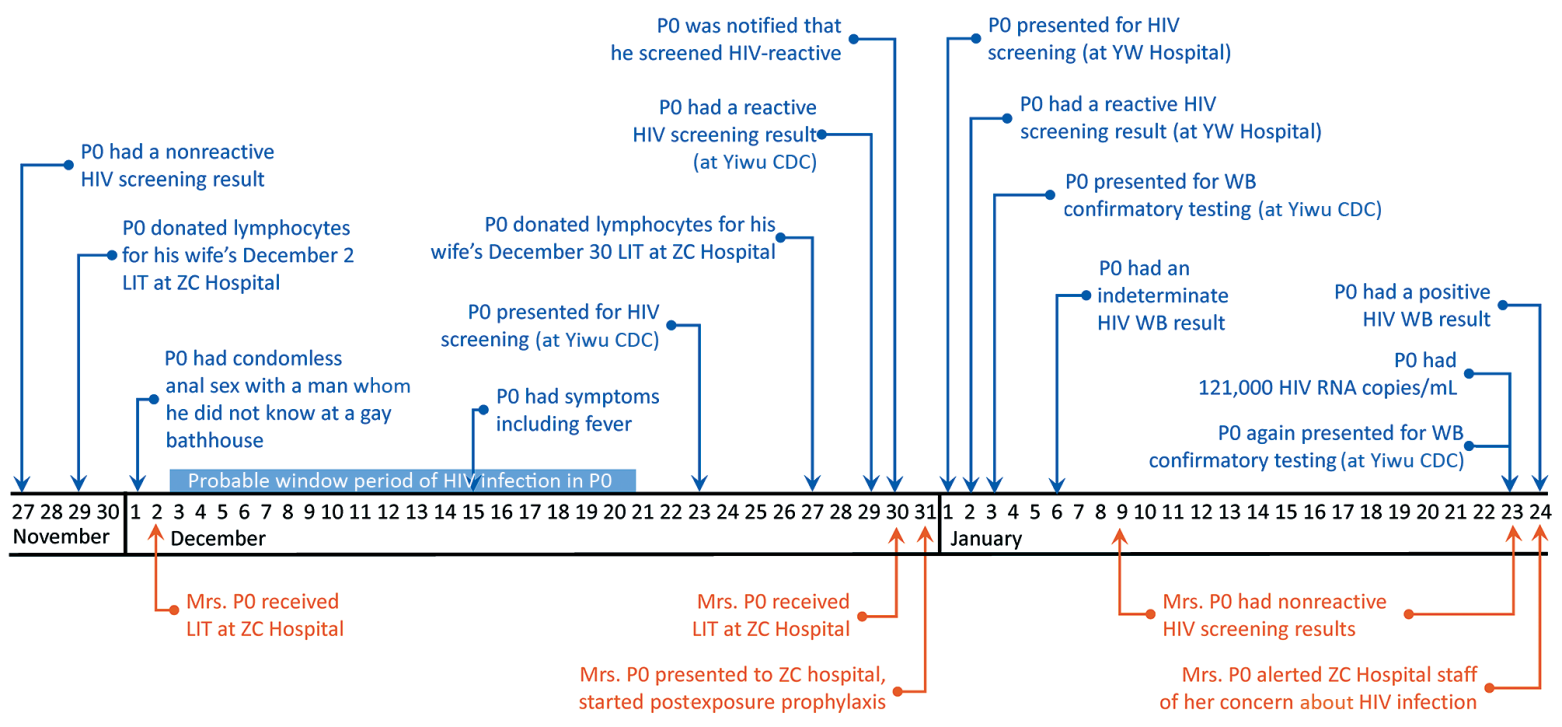
Timeline of HIV exposure and HIV diagnosis of the index case-patient, P0 (blue), and the HIV exposure of his wife, Mrs. P0 (orange), Hangzhou, China, November 27, 2016–January 24, 2017. CDC, Center for Disease Control and Prevention; LIT, lymphocyte immunotherapy; P, patient; WB, Western blot.

P0 was the 40-year-old husband of Mrs. P0. Public health workers constructed a 2-month timeline related to his HIV exposure and testing (Figure 1). His most recent negative HIV screening result was on November 27, 2016. The residual specimen was retested using a 4th-generation antigen/antibody test during the investigation and confirmed negative. The exposure event that probably led to P0’s HIV infection was traced to December 1, 2016, when he had condomless, receptive anal sex with a man he did not know at a gay bathhouse. Approximately 2 weeks later, fever developed, and he began to suspect HIV infection.

P0 went to the Yiwu CDC for HIV testing on Friday, December 23, but his specimen was not tested until December 29 (HIV testing by both antibody-only and antibody/antigen EIA is only performed on Thursdays at Yiwu CDC). He was not informed of his reactive result until noon on December 30, at which time he was encouraged to return for confirmatory testing. However, P0 instead sought rescreening at another facility. He returned for confirmatory testing on January 3, 2017. His first WB result was indeterminate (gp160/p24). His second result (using a new specimen obtained on January 23) was positive (gp160/gp120/p66/p31/p24), and his viral load (VL) was 121,000 copies/mL. P0 was informed of his HIV diagnosis on January 24 and started treatment the next day. He tested negative for HBV, HCV, and syphilis. Investigators determined that P0 made no other donations of fluids, cells, or tissues after his exposure on December 1, 2016.

### Laboratory Investigation

The audit of the LIT laboratory resulted in 5 main findings. First, although the appropriate protocol was used and requires that each disposable sterile tube for lymphocyte processing is used only once, the laboratory experienced a shortage of these tubes for 1 day on December 30. To provide LIT to 34 women, >136 tubes were needed (34 for moving lymphocytes from culture containers to washing plates and 102 for washing the 34 cultures 3 times each). Approximately 100 tubes were available on December 30. Second, instead of stopping and calling this issue to the attention of a supervisor, the laboratory technician processing donated lymphocytes for LIT on December 30 deviated from protocol and reused tubes. Deviations occurred in 2 procedures: tubes were used repeatedly for moving lymphocytes from culture containers to washing plates and for washing the lymphocyte cultures. Third, the technician failed to properly document the work performed and upon interview, admitted to reusing disposable tubes “a few times,” but could not remember how many times or for which couples. Fourth, no deviation occurred on December 27 that could have caused contamination during blood specimen collection, lymphocytes separation, or lymphocyte culturing. Finally, LIT was performed at ZC Hospital only ≈1 time each month. No LIT had yet been conducted during December 30, 2016–January 25, 2017. No evidence of these failures, or other failures that could have similarly resulted in nosocomial transmission of HIV, was found before December 30 or during December 31–January 25.

The auditors concluded that lymphocyte processing deviated from the protocol on December 30 and that the technician responsible contaminated an unknown number of patients’ prepared lymphocytes on December 30 with lymphocytes from the index case-patient. Thus, all patients who received LIT on December 30 should be tested as if they had potentially been exposed. Auditors recommended that all the women and their husbands be tested for HIV, HBV, HCV, and syphilis.

### Contact Tracing

**Figure 2:**
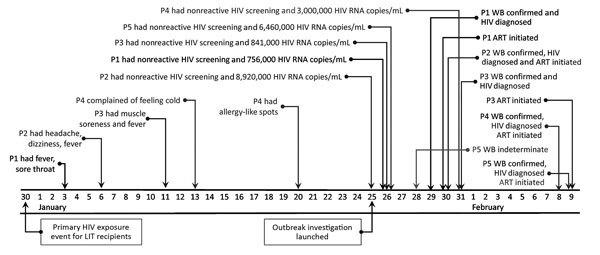
Timeline of HIV exposure, symptoms, diagnosis, and treatment initiation for the 5 HIV-infected women during nosocomial HIV outbreak, Hangzhou, China, December 30, 2016–February 9, 2017. P1, purple; ART, antiretroviral therapy; LIT, lymphocyte immunotherapy; P, patient; WB, Western blot.

Along with Mrs. P0, 33 other women received LIT at ZC Hospital on December 30. A medical records review found that all 33 had tested negative for HIV, HBV, HCV, and syphilis before beginning LIT in 2016. None reported any HIV risk behavior other than sexual contact, and none reported sexual contact with anyone other than their husbands. All 33 women (possible primary contacts) and their husbands (possible secondary contacts) were tested for HIV, HBV, HCV, and syphilis. Five cases of HIV were found (Figure 2).

#### Case-Patient 1

P1, age 35 (not pregnant) at the time of the investigation, received LIT at ZC Hospital on November 4, December 2, and December 30, 2016. She reported having fever, sore throat, and other symptoms, for which she had been given penicillin at a local clinic for a suspected bacterial infection. HIV serologic results were nonreactive, but virologic results were positive with VL of 756,000 copies/mL on January 26, 2017, suggesting acute infection. Later confirmatory WB result was positive (gp160/gp120/p41/p24). P1 was informed of her diagnosis on January 29, and she initiated ART the next day.

#### Case-Patient 2

P2, age 28 and pregnant (10 weeks’ gestation) at the time of the investigation, received LIT at ZC Hospital on July 22, August 19, September 16, October 14, November 11, December 9, and December 30, 2016. She reported symptoms including headache, dizziness, and fever but sought no care. HIV screening results were nonreactive, but virologic results were positive with VL of 8,920,000 copies/mL on January 25, 2017, suggesting acute infection. Confirmatory WB result was positive (gp160/gp120/p24). P2 was informed of her HIV diagnosis, and she enrolled in PMTCT and initiated ART on January 30.

#### Case-Patient 3

P3, age 34 (not pregnant) at the time of the investigation, received LIT at ZC Hospital on October 10, November 4, December 2, and December 30, 2016. She had muscle soreness and fever but did not seek care. HIV screening results were nonreactive; however, virologic results were positive with VL of 841,000 copies/mL on January 26, 2017, suggesting acute infection. Confirmatory WB result was positive (gp160/gp120/p24) on January 31, and she was informed of her diagnosis on the same day. She began ART on February 9, 2017.

#### Case-Patient 4

P4, age 28 and pregnant (17 weeks’ gestation) at the time of the investigation, received LIT at ZC Hospital on March 4, April 1, April 29, May 27, June 24, July 29, August 19, November 18, December 9, and December 30, 2016. She reported symptoms including feeling cold and red allergy-like spots on her chest but did not see a doctor. HIV screening results were nonreactive, but virologic results were positive with VL of 3,000,000 copies/mL on January 31, 2017, suggesting acute infection. First confirmatory WB result was indeterminate (gp160/p24); second was positive (gp160/gp120/p24). P4 was informed of her diagnosis, enrolled in PMTCT, and initiated on ART on February 2.

#### Case-Patient 5

P5, age 34 (not pregnant) at the time of the investigation, received LIT at ZC Hospital only on December 30, 2016. She reported no symptoms. HIV screening results were nonreactive, but virologic results were positive with VL of 6,460,000 copies/mL on January 26, 2017, suggesting acute infection. First confirmatory WB result was indeterminate (gp160), but the second was positive (gp160/gp120/p24). P5 was informed of her diagnosis and initiated ART on February 9.

#### Other Contacts

The remaining 29 women, including Mrs. P0, the wife of the index case-patient, all had multiple negative serologic and virologic results during follow-up (Table 1). None had HBV, HCV, or syphilis.

**Table 1:** Interval between HIV exposure and follow-up HIV tests for the 37 persons followed up after initially screening HIV-nonreactive in investigation of nosocomial HIV outbreak, Hangzhou,China, 2016–2017*

Potential contact	Days between HIV exposure and follow-up HIV tests							
1st test	2nd test	3rd test	4th test	5th test	6th test	7th test	8th test
Primary†								
Mrs. P0‡	24	29	45	68	103	130	191	464
Q1	28	48	82					
Q2	28	58	86	186				
Q3	27	29	59	84	182			
Q4	28	58	86	192				
Q5	28	45	86	192				
Q6	28	56	94	188				
Q7	27	28	57	99	211			
Q8	29	58	100	189				
Q9	27	57	45	85				
Q10	27	28	60	93	187			
Q11	28	64	106	202				
Q12	29	55	83	185				
Q13	27	29	58	89				
Q14	27	35	62	92	199			
Q15	27	33	64	93	212			
Q16	27	45	93					
Q17	27	33						
Q18	27	34	59	90	189			
Q19	27	33	63	91	187			
Q20	28	60	90	194				
Q21	30	55	80	192				
Q22	31	45	93	186				
Q23	31	53	81	188				
Q24	33	55	89	187				
Q25	32	63	92	196				
Q26	33	63	95	189				
Q27	33	47	97	193				
Q28	33	60						
Secondary§¶								
P3’s husband	7	30	57	97	182			
P4’s husband	11	28	52	98	180			
P5’s husband	2	18	25	32	60	196		
Infant P0	3	42	90	242				
Infant P2	3	43						
Infant P4	1	44						

*First HIV test was conducted using both nucleic acid testing and EIA. All subsequent HIV tests were conducted using EIA only. Blank cells indicate no further HIV test. EIA, enzyme immunoassay; LIT, lymphocyte immunotherapy; P, patient infected with HIV; Q, women who might have been exposed to HIV but were not infected. †Beginning of the time interval was counted from contaminated LIT at ZC Hospital on December 30, 2016. ‡Mrs. P0 is the wife of the index case-patient, P0. She is continuing to be followed once a year for at least 3 years since she discontinued antiretroviral therapy. Her most recent HIV test, on March 9, 2018, was again negative. §For husbands, beginning of the time interval was counted from the most recent sexual contact with the wife from the wife’s exposure on December 30, 2016, through the start of the investigation on January 25, 2017. Husbands received both serologic (4th-generation Ag/Ab EIA, Shanghai Kehua Bio-Engineering, Shanghai, China) and virologic testing (HIV-1 RNA, COBAS AmpliPrep/COBAS TaqMan HIV-1 Test v2.0, Roche, Branchburg, NJ, USA). ¶For infants, beginning of the time interval was counted as the date of birth. The first 3 HIV tests for infants were early infant diagnosis tests by a standard nucleic acid testing protocol. The fourth test for infant P0 was a 4th-generation Ag/Ab EIA.

Although all 33 husbands were tested initially, 3 husbands of the 5 women with newly diagnosed HIV infection, all of whom who reported sexual contact with their wives after December 30, 2016, were followed and provided HIV serologic and virologic testing, as well as HBV, HCV, and syphilis testing. All results were negative. Additionally, the infants of Mrs. P0 (born July 9, 2017), P2 (born August 14, 2017), and P4 (born July 3, 2017) were tested for HIV by early infant diagnosis (EID; by PCR) and were HIV-negative.

### Phylogenetic Investigation

**Figure 3:**
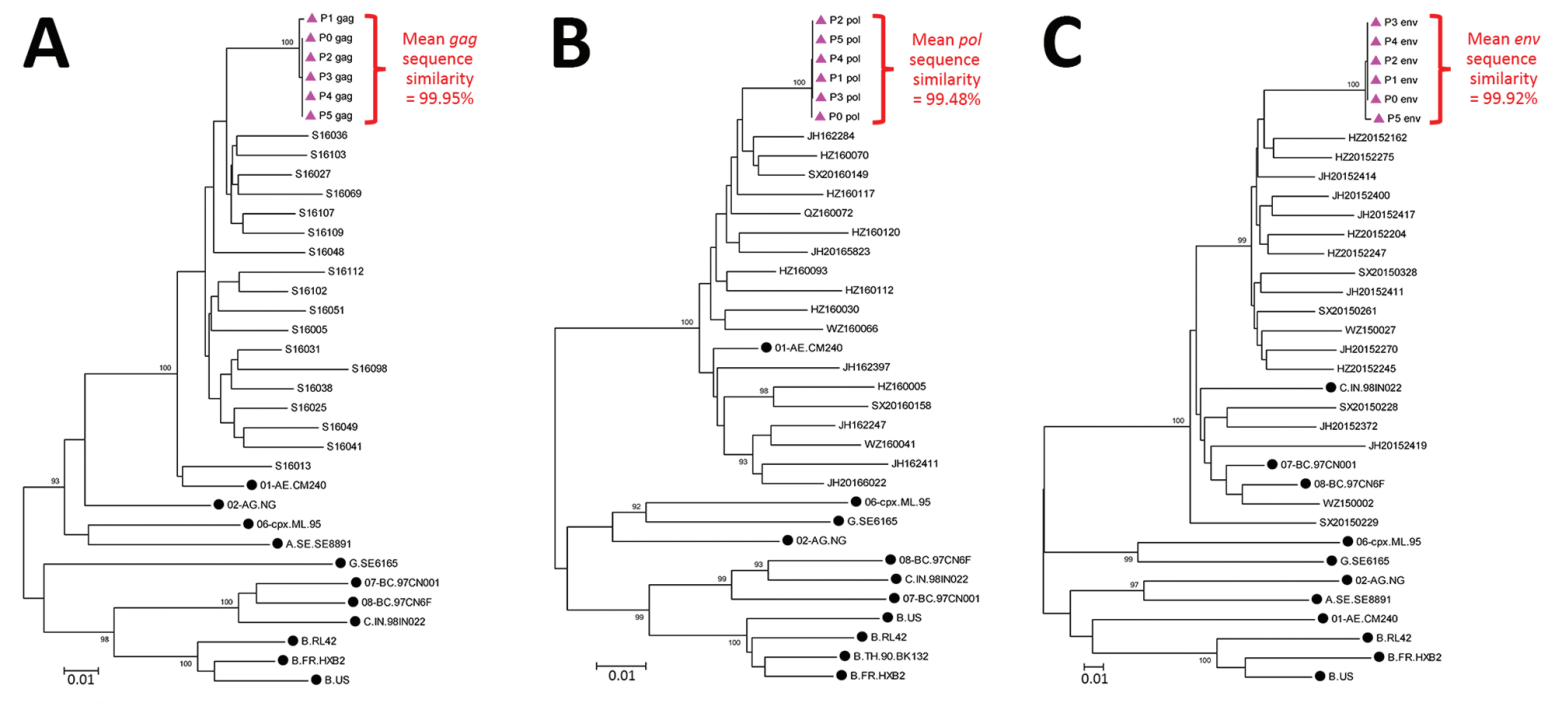
Phylogenetic trees showing relationships between HIV-1 gene sequences from index case-patient and 5 women infected during nosocomial HIV outbreak, Zhejiang Province, China, 2016–2017, and reference sequences. Bootstrap values >90% only are shown for *gag* sequences (A), *pol* sequences (B), and *env* sequences (C). Triangles indicate index case-patient (P0) and 5 women found to have HIV infection (P1–5); dots indicate international reference sequences. Scale bars indicate nucleotide substitutions per site. P, patient.

The HIV sequences derived from the index case-patient and the 5 women with newly diagnosed HIV infection shared a very high degree of similarity: mean of 99.95% for *gag*, 99.48% for *pol*, and 99.92% for *env* (Table 2). The *gag* and *pol* sequences were consistent with HIV-1 subtype CRF01_AE, and *env* sequences were subtype as C, indicating that all 6 persons were infected with a recombinant CRF01_AE/C strain. Phylogenetic trees of *gag*, *pol*, and *env* sequences (Figure 3) indicate a very close genetic relationship between the virus present in the 5 women with newly diagnosed infection and the index case-patient. The sequences of all 3 genes map to monophyletic clusters in 100% of bootstrap replicates with genetic distances of <0.015.

**Table 2:** Similarity of HIV genetic sequence of viral nucleic acid from the index case-patient and the 5 women infected by during nosocomial HIV outbreak, Hangzhou, China, 2016–2017

Region	Sequence similarity, %					
Case-patient 1	Case-patient 2	Case-patient 3	Case-patient 4	Case-patient 5	Mean
*gag*	99.70	100.00	100.00	100.00	100.00	99.95
*pol*	99.50	99.40	99.50	99.50	99.50	99.48
*env*	100.00	100.00	100.00	100.00	99.60	99.92

### Summary of Outbreak Response

In response to this outbreak, the National Health Commission immediately suspended all LIT services nationwide, and all 34 couples involved were provided counseling and support. The epidemiologic investigation found that a lymphocyte donor (P0) had become infected with HIV before donation on December 27 and that laboratory contamination occurred on December 30, which together caused 5 women to become infected with HIV. HIV phylogenetic investigation confirmed the causal relationship. All 5 women had initiated ART as of February 9, 2017, only 15 days after the investigation began. All remaining 29 women who initially screened nonreactive and the husbands of the 5 infected women were followed up for 6 months; no additional HIV infections were found. The 3 pregnant women were provided PMTCT; their newborn infants were followed up, and no HIV infection was found. The laboratory technician was sentenced to 2.5 years in prison. The hospital director, deputy director, and division chief accountable for the laboratory were dismissed. LIT services were suspended until a new guideline was issued on December 22, 2017 (*12*).

## Discussion

These epidemiologic and phylogenetic investigations used techniques similar to those used during HIV outbreak investigations including a famous case of a Florida, USA, dentist (*13**–**16*); several criminal cases (*17**–**20*); a prison outbreak in Scotland, UK (*21*); 2 nosocomial outbreaks (*22**,**23*); and a recent outbreak in Indiana, USA, associated with injection drug use (*24*). These investigations identified P0 as the index case-patient for this nosocomial HIV outbreak and demonstrate that deviation from protocol and lapse in infection control during LIT were the cause. We have yet to detect HIV infection in Mrs. P0, the wife of the index case-patient, suggesting that her immediate initiation of PEP might have averted infection. However, we are unable to definitively determine Mrs. P0’s HIV status because she has been followed for only 9 months since she discontinued ART, and evidence of viremic rebound nearly 30 months after ART cessation was observed in the case of a child in Mississippi, USA (*25**,**26*). These results underscore the critical importance of quickly investigating a suspected outbreak. Among the 34 women potentially exposed, only 5 acquired infections, and potential onward transmission of HIV to their husbands and infants was averted.

This study was subject to at least 2 limitations. First, follow-up HIV testing for the 29 potentially exposed women was voluntary, and some declined to have third and fourth HIV tests. For example, Q17 was followed up at 27 days (with nucleic acid testing and antigen/antibody EIA) and 33 days (antigen/antibody EIA only) and, although unlikely, it is possible that she had undetected HIV infection. Second, as noted, we were unable to definitively ascertain Mrs. P0’s HIV status within the scope of this study. Hence, HIV infection linked to this outbreak might not yet have been diagnosed.

The results of this outbreak investigation offer important lessons that China must not ignore. First, the unacceptably long process of HIV diagnosis in China directly contributed to this outbreak. Ample evidence of the substantial benefit of streamlining and accelerating China’s HIV care continuum already exists (*27**–**29*). However, although a rapid 1-visit testing, diagnosis, clinical staging, and ART initiation protocol has been adopted, China must accelerate the pace at which these changes are implemented if it is to avoid another, similar outbreak.

Second, China must implement more frequent and thorough training for medical professionals on the risks of nosocomial HIV transmission. The finding that Mrs. P0’s attending physician at XX Hospital was concerned enough to start her on PEP but not to alert public health officials and the finding that the laboratory technician did not consider reusing sterile tubes to be unsafe both indicate that education about the risks for nosocomial HIV transmission is still lacking. China must act quickly to fill this gap.

Finally, laboratories in medical settings must be placed under stricter controls. Immediate supervision and monitoring and thorough and frequent laboratory audits should be implemented immediately in China’s medical laboratories. A high level of vigilance in the medical laboratory setting is critical if China is to prevent similar future nosocomial outbreaks.
